# Simulation of normal and pathological gaits using a fusion knowledge strategy

**DOI:** 10.1186/1743-0003-10-73

**Published:** 2013-07-11

**Authors:** Fabio Martínez, Christian Cifuentes, Eduardo Romero

**Affiliations:** 1 CIM&Lab - School of Medicine, Universidad Nacional de Colombia, Bogotá DC, Colombia

## Abstract

Gait distortion is the first clinical manifestation of many pathological disorders. Traditionally, the gait laboratory has been the only available tool for supporting both diagnosis and prognosis, but under the limitation that any clinical interpretation depends completely on the physician expertise. This work presents a novel human gait model which fusions two important gait information sources: an estimated Center of Gravity (CoG) trajectory and learned heel paths, by that means allowing to reproduce kinematic normal and pathological patterns. The CoG trajectory is approximated with a physical compass pendulum representation that has been extended by introducing energy accumulator elements between the pendulum ends, thereby emulating the role of the leg joints and obtaining a complete global gait description. Likewise, learned heel paths captured from actual data are learned to improve the performance of the physical model, while the most relevant joint trajectories are estimated using a classical inverse kinematic rule. The model is compared with standard gait patterns, obtaining a correlation coefficient of 0.96. Additionally,themodel simulates neuromuscular diseases like Parkinson (phase 2, 3 and 4) and clinical signs like the Crouch gait, case in which the averaged correlation coefficient is 0.92.

## Background

Quantification of complex movements such as human locomotion is a fundamental step towards an objective characterization of particular patterns associated to a certain degree of a disease [1-3]. The gait is the result of complex interactions between several sub-systems: neuromuscular, musculo-tendinous and osteo-articular, which work together to generate the body dynamics that underlies the bipedal displacement [4,5]. In despite of the intensive research in biomechanics [6], robotics [7,8], medicine [9] and computer animation [10,11], the biological complexity has hindered a proper understanding of the locomotor system. This problem has been partially overcome in the clinical routine by a gait estimation inferred from the gait laboratory [9,12,13]. Usually, a physician or rehabilitation expert determines whether there exist pathological gait patterns using exclusively her/his expertise [4,14,15]. Overall, diagnosis is supported using statistical tests carried out on the acquired gait laboratory data [16-20], with an inherent high degree of variability. In consequence, development of gait models that provide a quantitative gait description has become important in the process of supporting physician decisions [4,9,14,21].

The main contribution of the present work is a human gait model that accurately describes a set of kinematic gait patterns, normal or pathological. The model fuses two important gait information sources: an estimated Center of Gravity (CoG) trajectory and heel paths learned from actual gaits. The global motion is governed by the CoG trajectory of a compass physical pendulum representation, coupled to a spring that emulates the muscle function. This trajectory is regulated by learned heel paths, while the remaining joint patterns are estimated using a classical inverse kinematic method. The models benefit is demonstrated by accurately simulating two different sorts of neuromuscular gaits: Parkinson and Crouch patterns. Finally, a human-like leg structure is animated with the obtained trajectories, allowing the clinician to interact with the model and facilitating the interpretation of an observational analysis.

Many models have been previously proposed for simulating the human gait, with different complexity levels, depending on the application area. A first group includes bipedal descriptions that exclusively use structural information so that they are able only to determine global relationships between muscles and joint angles. These models exploit the conceptual simplicity of mechanical systems such as the inverted pendulum or mass-springs [22-26]. Basically, these approximations provide a locomotion description from an energy standpoint, simulating the change from the kinematic to potential energy during the gait cycle. These models are devised to coarsely classify normal and pathological patterns [18,27]. However, a main drawback of these approximations is that about a 20*%* of the gait cycle, corresponding to the double stance phase, is completely eliminated. These physical models are useful in areas like robotics since they eliminate the dependence on a robust control mechanism. Nevertheless, they are very limited for medical applications because of their strong simplifications, missing relevant gait aspects such as the non-linearities introduced by the heel strike.

A second group of human gait models are capable of simulating muscles and tendons during the gait. These models have obtained better gait representations, introducing muscular information that is required from a clinical standpoint in terms of interpretability, i.e., specific activity of certain muscle groups in musculoskeletal disorders like hemiplegic movements. These models have introduced new elements to simulate the control and energy storage of the locomotion process. Specifically, some gait approximations include the Hill model as the base of the muscle representation [4,5,15], but with no relation between the muscle and the locomotor structure and hence without any clinical meaning [28]. In these approaches, each model accelerates a specific body segment, obtaining a simplistic simulation of pathological movements. Likewise, these models are not accurate enough to describe the complex interaction among different groups of muscles. In addition, they require a certain number of parameters that need to be tuned, with the consequent dependence on an expert knowledge. Scott Delp [10,29] introduced a computational strategy that combines the Hill muscle model and structural information, accomplishing realistic normal and pathological simulations, but again, with a high degree of subjectivity at tuning the model parameters. Currently, several approaches have used some control-based strategies, requiring relatively few data to simulate simple human structures and predicting new motions [30]. These approaches include a large number of degrees-of-freedom while joint force profiles remain subjected to a large number of constraints [9,11,19,27,31]. These methods approximate human control systems and simulate some neurological pathologies [15], but these strategies require specific information about each particular motion to be simulated and therefore they demand a high degree of interaction and prior knowledge [27]. Moreover, these methods necessitate a large group of experimental data to generate natural motions so that their clinical usefulness still remains very limited.

## Materials and methods

**Figure 1:**
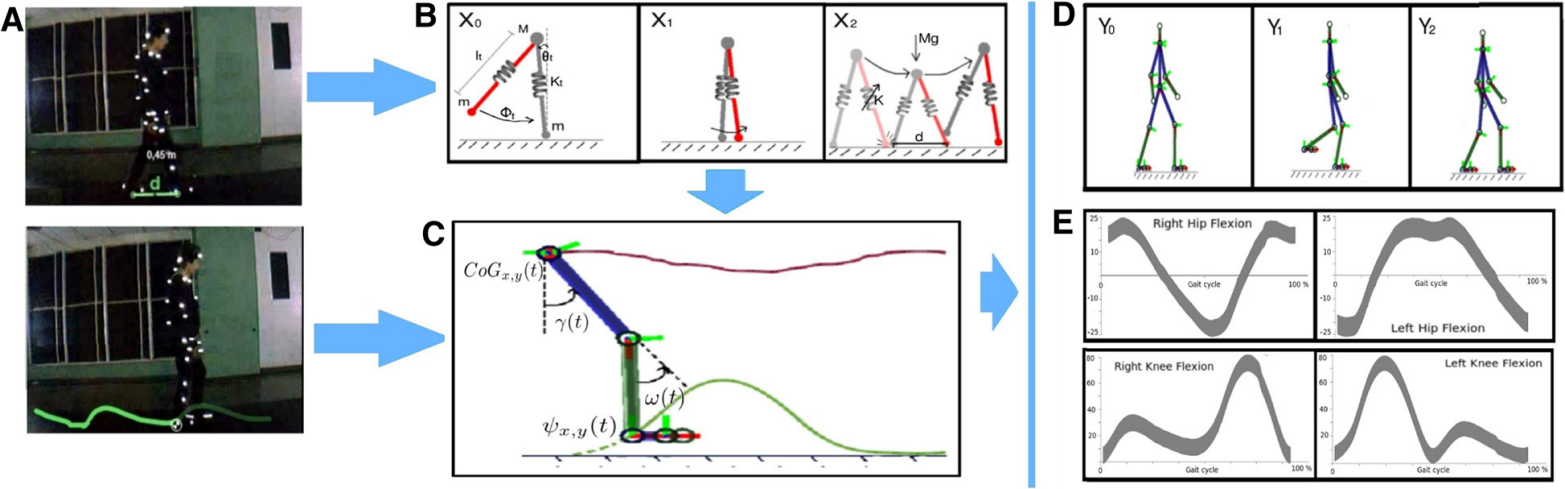
**Pipeline of the proposed model.** Firstly the *CoG*_*x*,*y*_(*t*) from the proposed physical model is computed (panel **(B)**). Additionally heel trajectories are learned for each kind of movement (panel **(A)**). Then, a fusion rule to compute kinematic patterns (Panel **(C)**) from both trajectories allows to simulate Normal and Pathological patterns (Panels **(D)** and **(E)**).

The present work simulates normal and pathological kinematic patterns by fusioning two important sources of information: a prior model of the *CoG*_*x*,*y*_(*t*) and real data trajectories. The proposed model is summarized in Figure 1. Firstly, the prior knowledge of the *CoG*_*x*,*y*_(*t*) is introduced using a physical gait model, a compass pendulum with springs coupled to both ends, representing the role of the knee and smooth tissues (see Figure 1(b) and a further description in section “*CoG*_*x*,*y*_(*t*) gait representation”). The inclusion of these non linear elements allows more accurate estimations of the *CoG*_*x*,*y*_(*t*). A second information source comes from actual heel trajectories that are used to regularize the estimated *CoG*_*x*,*y*_(*t*) and serve to simulate diverse pathological and normal motion (see Figure 1(a) and section “The fusion information strategy”). Additionally, this fusion facilitates an accurate estimation of the remaining joint trajectories, using a classical inverse kinematic framework. Finally the set of obtained trajectories animates a human-like leg structure that provides the clinician with a interpretable tool (see Figure 1(c-d)).

### Ethical approval

The study was approved by the Ethics Committees of the Institute. Written informed consent was obtained by the parents or, when applicable, by the patients.

### *CoG*_*x*,*y*_(*t*) gait representation

In human movement analysis, the gait is divided in cycles, coarsely classified as double and single stance phases [14,32,33]. The double stance period accounts for around 20*%* of gait cycle and stands for the body movement with both limbs touching the ground, while the single stance represents around 80*%* of gait cycle and corresponds to the interval in which a single limb supports the whole body weight. In this work the *CoG*_*x*,*y*_(*t*) for a complete gait cycle is approached using two complementary strategies: a compass pendulum for the single stance and a spring mass system for the double stance, as follows.

#### The single support phase

The single support phase conserves a regular periodicity which is properly captured using a compass pendulum representation. This strategy represents the upper part of the body by a mass *M* which moves forwards with respect to each fixed point (with mass *m*), describing a harmonic oscillating trajectory, similar to the inverted pendulum [22,34]. Likewise, the free foot swings with respect to this mass, establishing a simple pendulum pattern. Provided that these processes are coupled together, the human gait is modeled by a compass pendulum as two coupled non-linear differential equations:

(1) $$\begin{array}{l} \beta (1-\cos\phi )(3\ddot{\theta }-\ddot{\phi })\;-\;\beta \sin\phi ({\dot{\phi }}^{2}-2\theta \dot{\phi })\\ \;\;\;\;\;\;+\;(\frac{g\sin\theta }{l})(\beta (\sin(\theta -\phi )-1))=0\\ \ddot{\theta }(\beta (1-\cos\phi ))-\beta \ddot{\phi }+\beta {\dot{\theta }}^{2}\sin\phi +(\frac{\mathrm{\beta g}}{l})\sin(\theta -\phi )=0 \end{array}$$

where *β*=*m*/*M*, *θ* is the angle of the stance leg at the particular time *t* with respect to the slope and *ϕ* is the angle between the stance leg, and *l*_0_=*l*_*r*_=*l*_*l*_. This model also allows to simulate the swing foot when it hits the ground at the heelstrike, a time in the cycle that corresponds to *ϕ*(*t*)−2*θ*(*t*)=0 [34], when the double stance starts.

#### Double stance phase

Classical gait models often ignore the double support stance since they have been devised to simplify the gait rather than to accurately follow gait patterns. These simplifications have ended up by considering the leg structures as rigid segments, a hypothesis that easily leads to conclude for instance that the percentage of gait recovery is inefficient in energy terms, a reason why this phase has been eliminated in most of these strategies [4,9,21,35]. Additionally, important elastic contributions which produce relevant changes in the *CoG*_*x*,*y*_(*t*), during the double stance, are often neglected. These strong simplifications reduce an appropriate gait understanding and may lead to wrong interpretations when these models are used as supporting tools of clinical decisions.

A more accurate *CoG*_*x*,*y*_(*t*) description of the double stance phase was herein achieved by coupling a planar spring-mass system [36] to the compass pendulum, previously introduced. This change of the leg length *l* during the gait stance phase, allows to estimate the reaction force during the whole gait cycle, as illustrated in Figure 1 (B). Notice that each leg reaction forces points out towards opposite sides, separated by a distance *d* (the distance between the heelstrike and the other toe-off phase). The coupling is obtained as:

(2) $$\begin{array}{cc} M\ddot{x} & =l_{l}x-l_{r}(d-x)\\ \mathrm{Mÿ} & =l_{l}y+l_{r}y-\text{gM} \end{array}$$

where *g* is the gravity, *l*_*l*_ and *l*_*r*_ are the left and right legs, respectively and their length changes as:

(3) $$\begin{array}{cc} l_{l} & =k(\frac{l_{0}}{\sqrt{x^{2}+y^{2}}}-1)\\ l_{r} & =k(\frac{l_{0}}{\sqrt{{\left(d-x\right)}^{2}+y^{2}}}-1) \end{array}$$

These equations simulate the periodic vertical ground forces, with a period defined by $T=2\pi \sqrt{\frac{m}{k}}$. This independent formulation of each reaction force allows an independent analysis of each link, whereby gait abnormalities that asymmetrically affect each leg, such as the diplegia, can be simulated. Finally, the *CoG*_*x*,*y*_(*t*) is simulated by the integration of the two gait phases described as follows:

(4) $${\text{CoG}}_{x,y}(t)\;=\;\left\{\begin{array}{cc} l_{0}\left[\sin\theta (t),\cos\theta (t)\right] & \text{if}\phi (t)\;-\;2\theta (t)\;<\;0;\\ \left[\frac{l_{l}\frac{x^{3}}{6}-l_{r}(d-\frac{x^{3}}{6})}{M},\frac{l_{l}\frac{y^{3}}{6}+l_{r}\frac{y^{3}}{6}-\text{gM}}{M}\right] & \;\text{elsewhere}. \end{array}\right)$$

### The fusion information strategy

Although the *CoG*_*x*,*y*_(*t*) is a fundamental clinical descriptor [18], a useful identification of a particular disorder also requires a proper gait analysis of other anatomical joint trajectories. Accordingly, a more complete gait description was herein achieved by the fusion of two important sources of information: the physical gait strategy previously described and the learned heel trajectories.

The learned heel trajectories were modeled as a set of normal distributions with mean *μ*_*i*_ and variance $\sigma_{i}^{2}$ from three different groups of patients captured in a gait laboratory as:

$$\psi_{x,y}(t)=\overset{I}{\underset{i=1}{\sum }}w_{i}N(t|\mu_{i},\sigma_{i}^{2})$$

where *I* represents the total number of learned gait movements (normal, Crouch and Parkinsonian gaits). Each gait distribution was computed from 30 gait cycles belonging to 10 patients (7 men and 3 women). From this multi-gaussian distribution model, we can select a heel trajectory *i* to regularize the *CoG*_*x*,*y*_(*t*) associated to a particular gait movement. Likewise, the normal motion distribution allows a large variety of gait patterns of the same pathology. New relationships are inferred from these two trajectories by assuming the knee joint position as $r_{x,y}=\frac{l_{0_{x,y}}}{2}$[37]. Afterward, a classical inverse kinematic method is adapted to obtain two main kinematic patterns: the flexion-extension patterns of the hip *ω*(*t*) and knee *γ*(*t*). For doing so, at each time *t* of the gait cycle, a CCD method performs an iterative rigid transformation over each couple of joints. The two patterns are defined as:

(5) $$\gamma (t)=\text{acos}\left(\frac{{\text{CoG}}_{x,y}{\left(t\right)}^{2}-r_{x,y}^{2}-r_{x,y}^{2}}{2}\right)$$

(6) $$\omega (t)=\text{atan}2(\psi_{x,y})+\text{atan}2(r_{x,y}\sin\gamma ,r_{x,y}+r_{x,y}\cos\gamma )$$

where *r* is the distance between the *CoG*_*x*,*y*_(*t*) and *ψ*_*x*,*y*_(*t*). Unlike other approaches, this model estimates kinematic patterns with medical meaning, but the model can also obtain energy and ground force patterns for normal and pathological cases, obtained from the *CoG*_*x*,*y*_(*t*).

### Building up a human leg structure

**Figure 2:**
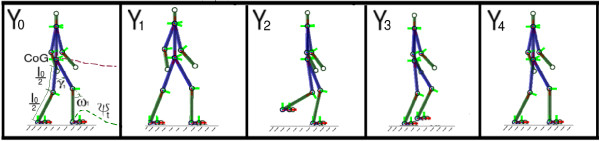
**The Figure shows the human-like leg structure used to simulate the set of kinematic patterns.** It can be used as software tool.

Finally, a human-like leg structure is animated using the set of kinematic patterns described above. This structure may be used as a clinical interpretability tool. For doing so, we define a human structure composed by a set of 12 rigid elements, connected together, as shown in Figure 2. The lower limbs follow a dynamics established by the proposed model, while the upper limbs are normal trajectories computed from real data of the gait laboratory.

### Modeling pathological movements

The proposed model is also capable of simulating pathologic patterns such as the spastic diplegia (typically represented by a Crouch Gait) and Parkinson, an advantage with respect to other classical models.

Firstly, the model is used to simulate a Crouch gait. This motion is produced by a neuro-muscular disorder known as the spastic diplegia that is characterized by the presence of muscle rigidity and loss of muscle force, affecting predominantly legs, arms and face. The clinical signs include gait pattern distortions of the sagittal plane, like bent-rigid knees, flexed hips and certain anatomical changes like lumbar lordosis. Such signs were herein modeled by setting the spring constant to values close to the estimated leg springiness. The resultant kinematic patterns are thus related with an increase in the energy consumption, showing a flexion rigidity of the hip and knee. The model is also used to simulate typical Parkinson gait patterns. In this case, the gait patterns are produced by a degenerative disorder of the central nervous system and are characterized by rigidity and slowness of the human movements: this gait is characterized by short steps. These Parkinsonian gait features were captured by fixing the *d* and *k* parameters, associated to the step length and the the knee flexion-extension, respectively. This representation results directly related to the energy consumption since in this case a particular displacement demands more energy than that required during a normal gait. Likewise, the kinematic gait patterns are characterized by a higher frequency than the observed for a normal gait. This kind of patterns can be modeled and simulated to approximate different phases of the disease, allowing thereby to objectively characterize the pathology.

**Figure 3:**
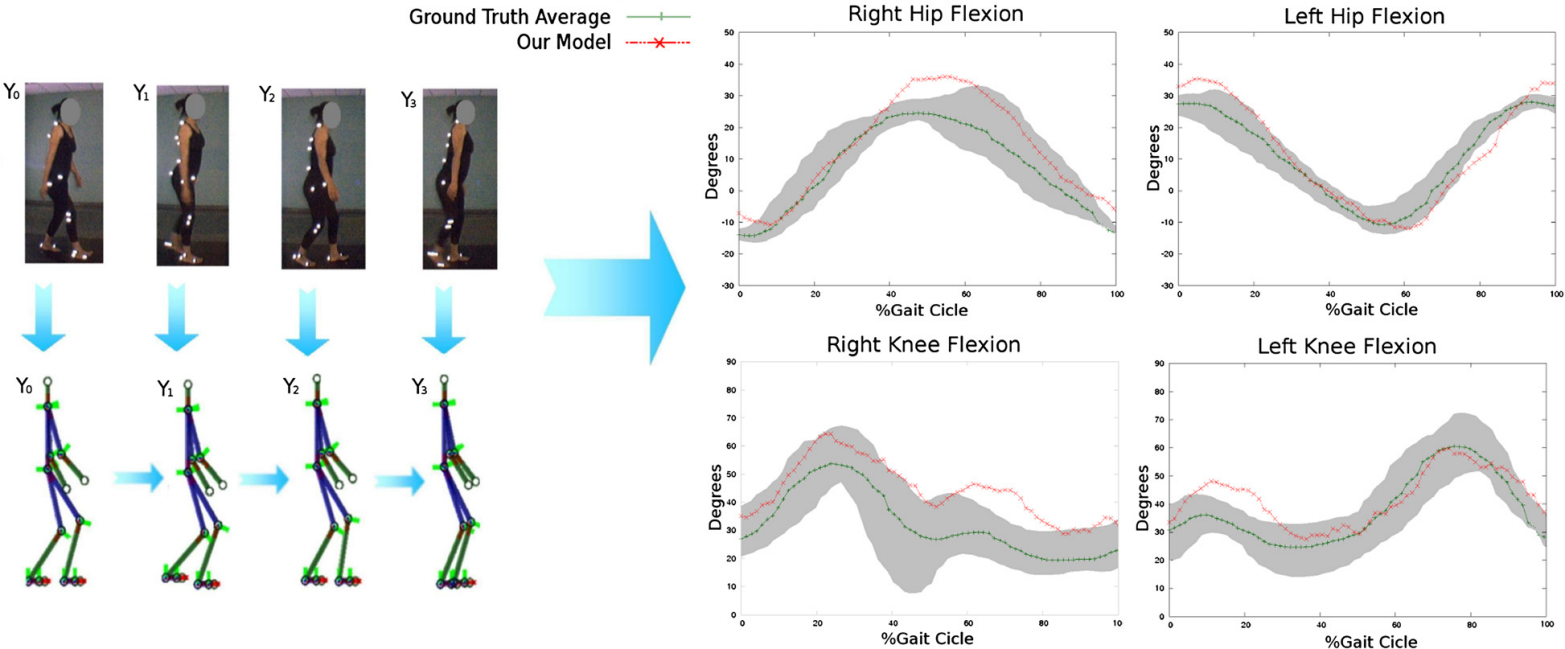
**Simulation of Crouch gait patterns.** In the left panel it is illustrated a sequence of human poses obtained from the proposed model. In the right panel it is presented the joint angle trajectory (red starred line), obtained with the proposed fusion strategy and compared with the ground truth patterns (shadowed gray zone whose mean trajectory is represented by the green line).

**Figure 4:**
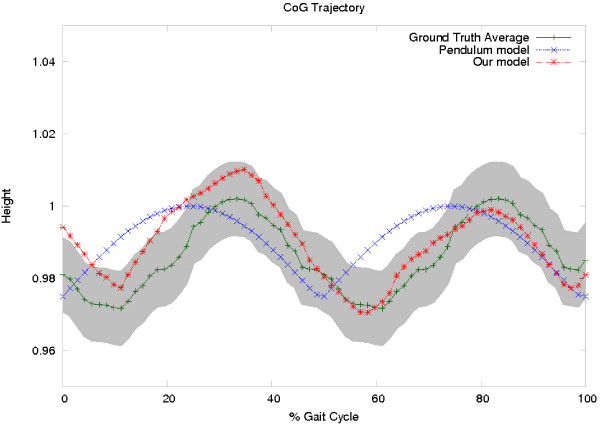
***CoG***_***x*,*y***_**(*t*) trajectory obtained with two different approaches.** The shadowed gray represents the normal distribution pattern for the *CoG*_*x*,*y*_(*t*) trajectory, whose mean *μ* is represented by the dark green line. The red starred line stands for the *CoG*_*x*,*y*_(*t*) trajectory obtained with our physical model while the blue crossed line represents a trajectory computed from a classical pendulum model.

Simulation of these pathologies requires to set the *l*_0_, *k* and *d* parameters, using actual patient data. For the Crouch gait simulation, the spring constant was fixed within a range of *k*=350 to 400, the *d*=0.65 and the *l*_0_ length was reduced to 5*%* of the initial 1 m, according to well known biomechanical parameters [1-3,20]. For the Parkinsonian gait simulation the *d* parameter was obtained from actual data and set to 0.58 *m*, the *k* was set to 500 and *l*_0_=1 meter. The heel paths fitted a normal distribution and were learned from actual patient data, as previously explained. The simulated trajectory precisely follows the different components of the abnormal gait pattern, in particular the flexed knees, the *CoG*_*x*,*y*_(*t*) attenuation and the step length, as shown in Figures 3 and 4 and reported in next section.

## Evaluation and results

Evaluation was carried out by comparing the estimated gait kinematic patterns with ground truth trajectories, of normal and pathological patterns, as reported in [2,14,21]. A quantitative evaluation was performed by calculating the correlation coefficient and the Fréchet distance between both trajectories, which are composed of temporal *x* and *y* paths, belonging to a single gait cycle.

**Figure 5:**
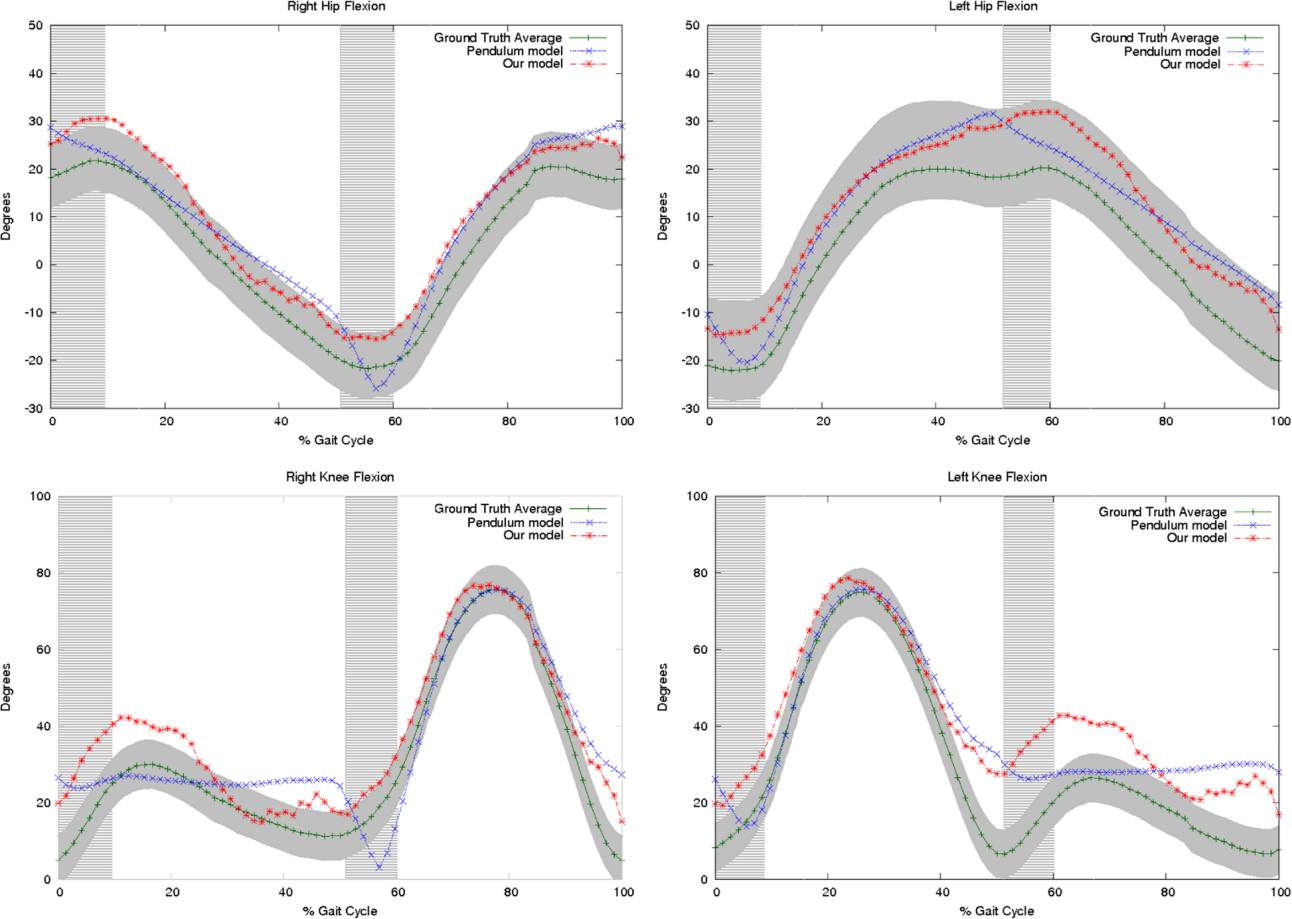
**Simulations for different gait patterns.** Panels A and B show the right and left Hip Flexion respectively. Panels C and D show the right and left knee flexion. The shadowed gray zone corresponds to the normal distribution of the possible joint angle trajectories and the dark green line represents the mean, i.e., the ground truth. The vertical shadowed green zone is the heel strike phase. Notice that the proposed model (red starred line) tracks better the ground truth, above all on the zones defined by the heel strike which are the most important when assessing pathological patterns. The blue crossed line corresponds to the trajectories computed from the fusion strategy but using the *CoG*_*x*,*y*_(*t*) of a classical compass pendulum.

A first part of the evaluation consisted in determining the *CoG*_*x*,*y*_(*t*) relation of two models, the physical model herein proposed and a classical compass pendulum model w.r.t the ground truth [38]. Figure 5 shows the decomposed motion for both models: the *x* axis representing the percentage of the gait cycle and the *y* axis the vertical displacement with respect to the body height, also in percentage. Both models follow a *CoG*_*x*,*y*_(*t*) periodic sequence, but the classical compass pendulum model systematically misses the discontinuity introduced by the heel strike, much more important in the *x* axis, while the proposed model accurately predicts this part of the cycle. Notice that the heel strike of the contralateral foot (toe off) actually occurs at about a 60*%* of the gait cycle.

**Figure 6:**
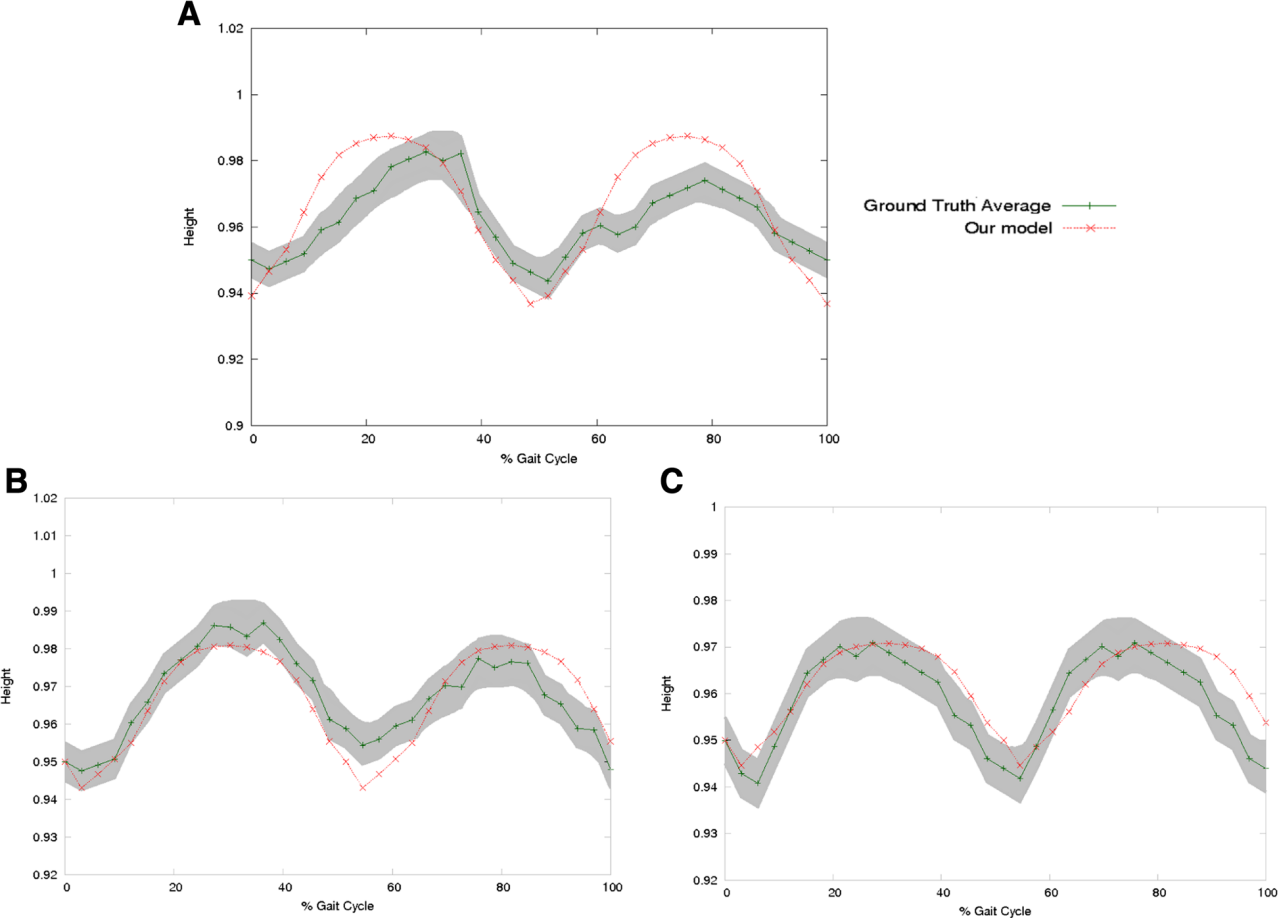
**Examples of the proposed method performance when estimating the dynamics of the CoG movement in different stages of the Parkinson Disease: Second Stage (A), Third Stage (B) and Fourth Stage (C).**

A second part of the evaluation compared the hip *γ*(*t*) and knee *ω*(*t*) joint-angle patterns of simulated normal gaits and ground truth patterns. For this assessment, the fusion strategy used two different *CoG*_*x*,*y*_(*t*) estimations: the proposed physical model and the classical compass pendulum previously described. The hip *γ*(*t*) and knee *ω*(*t*) patterns were expressed as joint-angle variations at the *y*-axis and plotted against the gait percentages at the *x*-axis, previously weighted by the entire duration of a cycle. Figure 6 shows the ground truth and the predicted gait patterns for a sagittal view (right and left) of a complete cycle, using the two *CoG*_*x*,*y*_(*t*) estimations. The joint-angle trajectories computed from the fusion strategy show a very close Correlation Coefficient (CF) w.r.t the ground truth patterns (CF =0.8 using the compass pendulum, CF =0.9 using the herein proposed physical approach). During the single stance phase, the angle trajectories computed from both CoG paths have a high correlation, nevertheless the conventional pendulum misses about a 40*%* of the angle variation because of the nonlinearity introduced by the heel strike and therefore the curve correlation also falls down. In contrast, the joint-angle trajectories obtained from the fusion strategy with the proposed physical model follows the actual gait paths and its correlation coefficient remains larger than 0.8. Significant differences were then reported with the conventional compass pendulum, specifically for the part of the cycle dominated by the heel strike.

Table 1 shows the correlation coefficient obtained with the temporal differences between both joint angle estimates and the ground truth. This measure was applied only to those gait segments associated with the heel strike since it was previously confirmed that performance in the other gait phases are comparable because both models are based in the pendulum principle to represent the single stance phase.

**Table 1:** Correlation factor computed for a normal Gait using two different physical models

**Pattern**	**R.Hip**	**R.Knee**	**L.Hip**	**L.Knee**	**CoG**
Garcia’s Model	0,17 ±0.02	0,38 ±0.05	-0,02 ±0.01	0,17 ±0.05	0,52 ±0,01
Our Model	0,89 ±0.02	0,99 ±0.01	0,95 ±0.003	0,99 ±0.02	0,84 ±0,01

Differences were found to be significant during the heel strike phase (student t-test, *p*<0.05) for the joint angle paths computed with the conventional pendulum *CoG*_*x*,*y*_(*t*), while the joint angle trajectories estimated with our physical model, obtained correlations of about 96*%*. In contrast, joint angle paths computed with the conventional pendulum *CoG*_*x*,*y*_(*t*) obtained barely correlations of about 46*%*, evidencing the weakness of this type of models during this gait phase.

On the other hand, since the whole problem consists in following temporal series which are highly non-linear and whose dynamics is therefore very difficult to establish, evaluation should also include a type of measure that determines a level of agreement between two trajectories. We have then measured this level using the Fréchet distance between two temporal series, i.e., the ground truth and any of the joint-angle trajectories obtained from the fusion strategy using the two models. Briefly, the Fréchet measure estimates how close two trajectories are during the temporal capture, that is to say it estimates how similar these two curves are. Two trajectories are then similar if this distance is close to zero, the smaller this distance the closer the curves are. The Fréchet distance between two curves is the length of the shortest path between two points that are simultaneously moving through the two curves. The Fréchet metric uses a particular direction of the two curves because the pairs of points whose distance contributes to the Fréchet distance sweep continuously along their respective curves. This makes the Fréchet distance a better measure of similarity for curves than alternatives, such as the Hausdorff distance, for arbitrary point sets. It is possible for two curves to have a small Hausdorff distance but large Fréchet distance.

Table 2 shows the Fréchet measure obtained from temporal differences between estimates and ground truth. Again, the *CoG*_*x*,*y*_(*t*) was assessed as well as the hip *γ*(*t*) and knee *ω*(*t*) joint-angles, showing smaller differences with our model. Interestingly, the close curve similarity between the joint angle trajectories estimated from our *CoG*_*x*,*y*_(*t*) and the ground truth, achieved a gain of 20*%* with respect to the classical compass pendulum model, that is to say, joint angle trajectories computed from our physical model were about 20*%* more accurate.

**Table 2:** Fréchet distance for a normal gait using two different physical models

**Pattern**	**R.Hip**	**R.Knee**	**L.Hip**	**L.Knee**	**CoG**
Garcia’s Model	0,31295 ± 0,025	0,30765 ± 0,023	0,37695 ± 0,017	0,35305 ± 0,025	0,0192 ± 0,0024
Our Model	0,18475 ± 0,012	0,14 ± 0,018	0,26515 ± 0,009	0,21635 ± 0,014	0,0132 ± 0,0041

Finally, a third part of the evaluation focused on challenging the fusion strategy to simulate pathological patterns. A Chrouch gait was tracked by changing the value of the *k* constant. Figure 3 shows a typical cycle obtained with the proposed model when tracking this pathological movement. It is observed in this illustration a close relationship between the trajectory described by the proposed model and the pathological pattern. A useful clinical evaluation requires a precise track of the consecutive ups and downs described by this pattern, rather than the magnitude changes.

The Crouch gait simulation was also compared with the two similarity metrics previously introduced, i.e., the correlation factor and the Fréchet distance, as illustrated in Table 3. The correlation factor is larger for upper joints, as expected since movement is much smaller, but yet correlation is high with joints such as the knees.

**Table 3:** Correlation factor and Fréchet distance when simulating the Crouch gait with the proposed fusion strategy

**Metric**	**R.Hip**	**R.Knee**	**L.Hip**	**L.Knee**	**CoG**
Correlation factor	0,96 ± 0,01	0,88 ± 0,04	0,96 ± 0,01	0,93 ± 0,03	0.92 ± 0,02
Fréchet distance	0,19 ± 0,006	0,26 ± 0,001	0,13 ± 0,004	0,21 ± 0,002	0,29 ± 0,003

The proposed approach was also used to simulate the Parkinsonian gait in different stages of the disease. For each Parkinson stage it was computed the most probable *k* and *d* parameters (See Table 4). Then, using these parameters it was generated a *CoG*_*x*,*y*_(*t*) with the proposed physical model (Figure 4).

**Table 4:** Model parameters learned from 10 patients in different stages of the Parkinson disease

**Model Parameter**	**Stage 2**	**Stage 3**	**Stage 4**
k average	52.3 ± 6.22	67.42 ± 8.25	96.8 ± 2.54
d average	0.76105 ± 0.087	0.5576 ± 0.0474	0.4356 ± 0.0294

**Figure 7:**
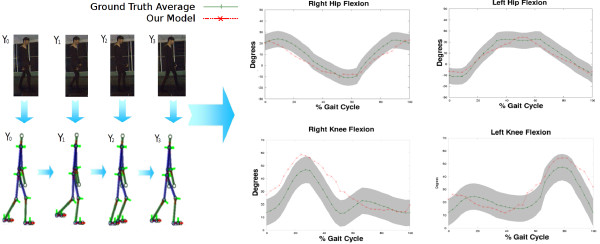
**Simulation of Parkinsonian gait patterns.** In the left panel it is illustrated a sequence of human poses obtained with the structural model, corresponding with actual patient poses. In the right panel it is presented the joint angle trajectory (red starred line) obtained with the fusion strategy and compared with a ground truth patterns (shadowed gray zone whose mean trajectory is the green line).

The rule of fusion, with the computed *CoG*_*x*,*y*_(*t*) and the learned heel paths from groups of patients, was used to compute the hip *γ*(*t*) and knee *ω*(*t*) joint-angle trajectories. The set of these trajectories allows to animate an articulated model as a virtual representation that shows characteristic Parkinsonian signs such as the short step and the slight flexion of the knee and the hip, as illustrated in Figure 7. Each Parkinson disease stage was also compared with the two similarity metrics, previously introduced, i.e., the correlation factor and the Fréchet distance, and results are summarized in Table 5. These results demonstrate that our fusion strategy simulates accurately different Parkinsonian gaits stages. The angular joint variations are very similar w.r.t to real patterns, obtaining a CF higher than 0.90 while the CoG achieves an accuracy larger than 0.80. The performance for every Parkinson level was similar, showing a compact representation for the gait with our method. Additional files 1, 2 and 3 show examples simulating the three different gates previously mentioned: the Normal, Crouch and Parkinsonian and their corresponding kinematic hip and knee flexion-extension patterns.

**Table 5:** Results obtained simulating the Parkinsonian gait for in each stage of the disease considered in this work

**2nd Stage**	**Metric**	**R.Hip**	**R.Knee**	**L.Hip**	**L.Knee**	**CoG**
	Correlation factor	0.92 ± 0.008	0.91 ± 0.01	0.95 ± 0.008	0.93 ± 0.009	0.83 ± 0.004
	Fréchet distance	0.09 ± 0.001	0.11 ± 0.002	0.09 ± 0.001	0.12 ± 0.002	0.12 ± 0.001
**3rd Stage**	**Metric**	**R.Hip**	**R.Knee**	**L.Hip**	**L.Knee**	**CoG**
	Correlation factor	0.95 ± 0.012	0.89 ± 0.009	0.93 ± 0.012	0.96 ± 0.01	0.88 ± 0.003
	Fréchet distance	0.09 ± 0.001	0.13 ± 0.002	0.09 ± 0.001	0.08 ± 0.002	0.13 ± 0.016
**4th Stage**	**Metric**	**R.Hip**	**R.Knee**	**L.Hip**	**L.Knee**	**CoG**
	Correlation factor	0.93 ± 0.008	0.90 ± 0.008	0.96 ± 0.008	0.91 ± 0.008	0.86 ± 0.005
	Fréchet distance	0.09 ± 0.002	0.12 ± 0.001	0.17 ± 0.002	0.13 ± 0.001	0.04 ± 0.001

## Discussion

The gait can be thought of as a sequence of complex combinations of several subsystems that help the body to keep the balance while it gains support and propulsion [39]. The gait analysis aims to interpret the complex combination of several motion patterns generated by the interaction of different systems. In the clinical routine, the gait examination is considered as the most important tool for identifying motion disorders, and it is also used as a biomarker of some neuromuscular illnesses like the cerebral palsy or the Parkinson disease, supporting thereby diagnosis and follow up. Interestingly, during this analysis it is possible to evaluate the effectiveness of a specific treatment and the particular response of the multiple gait subsystems. However, these analyses are not actually carried out in the clinical practice, among others because this requires a complete correlation of all pattern recorded, which is very time consuming and examiner dependent. Moreover, measures are contaminated by noise during the capture or, by invasive devices like markers, which inevitably alter the natural motion gestures. Overall, most clinical examinations are devoted to capture a general gait picture which makes that treatments are globally addressed. The actual utility of devising gait models is that they may improve understanding of some subsystem patterns from the captured data, even if they are noisy, and hence they allow to plan more specific treatments.

This work has presented a new fusion scheme that simulates a large set of gait patterns, including pathological conditions. The model allows to identify the role of certain subsystems during the simulation, an important step towards planning oriented treatments. This scheme uses two important information sources, i.e., kinetic and kinematic components, maintaining the possibility of an energy consumption analysis. The fusion method assumes that the gait kinematic patterns must follow basic physical principles. The underlying trajectories are generated by an adapted version of the compass pendulum representation which has been extended with elements that store energy, describing a larger number of abnormalities than it has been possible so far. These trajectories are then regularized by actual learned paths, completing thereby the rule of fusion by means of an inverse kinematic approximation. A broad range of gait pathologies can thus be emulated with the proposed approach, mainly those characterized by flexion-extension restrictions or leg stiffness, as for instance patients with diabetes mellitus (DM) and peripheral neuropathy (PN) who commonly present very short stride lengths, slower walking velocities and unstable upright postures [40,41]. Likewise, these simulations may be extended to gait pathologies that compromise knee dorsiflexors and extensors like the steppage gait.

The models reported in the literature are very limited when describing particular variations of a pathological motion. Gait models can be coarsely divided in two large groups: physical based models and musculoskeletal representations. Physical models use an inverted pendulum or a spring mass system that allow an energy consumption analysis and a global dynamic description. These models nevertheless fail to mimic pathological patterns because of the strong simplifications and restrictions, i.e., pendular models represent only the single stance while the double stance is completely omitted. Musculoskeletal representations, typically more complex than physical-based models, are able to simulate muscle patterns at each gait phase by adding some non linear muscle-tendon interactions, traditionally modeled by the Hill’s model or prior information coming from data obtained from actual gait laboratories. Currently, musculoskeletal models associate each leg segment to a Hill’s model. However, a main drawback of this approach is that there is no interaction between the different segments and hence simulations are quite far from experimental data, therefore, missing any anatomical meaning [10,29]. Best performances are obtained by combining a prior model with observations coming from actual data [4,5,10,15], but these models completely neglect important kinetic relationships and require a high level of expertise to properly tuning the model parameters. In contrast, physics based models are simple and usually tuned with a small number of variables. This simplicity, nonetheless, leads to most physics based models to miss important phases of the gait cycle. The fusion model proposed in this work represents the single stance by an articulated double pendulum system, while the double stance is properly simulated by a spring mass component that stands for the important knee motion contribution. The proposed model in addition emulates a whole skeleton structure by animating this architecture from the CoG trajectory. The whole strategy allows a natural simulation of non-linear gait patterns, representing several kinds of movements. Evaluation demonstrated the fusion model advantages, by comparing several kinematic patterns like the CoG trajectory and the hip and knee flexion-extension movements, considered as the most representative gait patterns for determining whether a motion is pathological or not [1-3].

The first evaluation compared the adapted physical model with a classical compass pendulum. An appropriate extraction of the CoG is essential since this biomarker is an efficient indicator of the normal/abnormal gait pattern, it constitutes one of the most important markers in pathologies such as hemiplegia, paraplegia or dystonia. A pathological gait can be analyzed in terms of energy using the CoG, which tracks the transfer of potential to kinetic energy (recovery), i.e. normal gait patterns loss 40% of their energy in this transfer, a higher loss is pathological [42]. The CoG trajectory described by our physical model achieved a CF of =0.84 while classical pendulum model only achieved a CF of =0.52, as illustrated in Figure 5.

In a second evaluation, hip and knee joint-angle paths were compared during the linear part of the cycle. Results showed a close correlation of the fusion strategy with respect to gold standard patterns, a CF of =0.9 for our strategy and a of CF =0.8 for trajectories computed from the classical compass pendulum. The same test was repeated exclusively for the nonlinear part of the cycle: a CF of =0.96 was estimated with the fusion model while a CF of =0.35 was computed with the classical compass pendulum. Likewise, while the complete proposed strategy obtained a 0.1 Fréchet distance, the joint trajectories estimated with the classical pendulum yielded a 0.35 Fréchet distance for the normal gait. Overall, the largest error was obtained during the nonlinear parts of the gait cycle like the heelstrike and the hip moments. This fact illustrates the relevance of obtaining a complete CoG global description even to estimate the remain joint angle trajectories

When simulations were performed for pathological patterns, the fusion method maintained in average a CF =0.90. Two neuromuscular disorders were simulated: the cerebral palsy and the Parkinson disease in three different stages. For both abnormal movements, actual patient motion data allowed to adjust parameters and to obtain closer CoG trajectories.

During the Crouch gait simulation, the model parameters were set to *k*=400 and *d*=0.65, according to the data learning process. Simulations achieved a CF of =0.9, a very close representation of actual patterns (Figure 6). The Fréchet distance maintained a comparable performance when tracking the ground truth. The Parkinson simulation, in three different disease stages, also shows a very alike representation w.r.t actual patterns. The model parameters were fixed according to Table 4 for each stage disease. The fusion model achieved in average a CF =0.92 and a Fréchet distance average of 0.1, demonstrating the close likeness of the patterns obtained and actual data. These results demonstrated the model ability to accurately follow a different sort of gait patterns, either normal or abnormal. The model may be used as training tool for physician and also to predict the performance of a particular gait treatment.

Finally, it is worth to strengthen out the simplicity of the presented model and its ability to represent pathological movements by only tuning two parameters and with a relatively simple fusion strategy. The model may be used as a complementary tool of the classical gait analysis to determine an illness degree of any subsystem, either by correlating the compromise of any leg segment with the clinical data, or by perturbing the model and therefore the resultant trajectory.

## Conclusions and perspectives

This work has presented a fusion model to simulate normal and pathological kinematic gait patterns. Two main contributions are introduced in this work, a fusion strategy of two important information sources which allows the accurate estimation hip and knee joint angle trajectories. Additionally, a physical that describes the COG trajectory using a pendular motion for the single stance and a spring mass system for the double stance. The model is complemented by an animated structure that allows to visualize and quantify different gait patterns, i.e., the hip and knee flexion-extension. The proposed approach can be easily extended to simulate other pathologies or even to find more dynamic gait relationships that describe a particular movement. Finally the proposed model opens up an actual possibility towards understanding more complex gait phenomena, crucial in many applications of the prostheses design such as the alignment or the relationship between those prostheses and the different muscle subsystems.

## Competing interests

The authors declare that they have no competing interests.

## Authors’ contributions

FM developed the algorithms and evaluated the results of the model. CC developed the algorithms and evaluated the results of the learning algorithm. ER conceived the study, developed the fundamental ideas underlying this model, participated in the experimental design and was the director of the whole project. All authors read and approved the final manuscript.

## Supplementary Material

Additional file 1 **Simulation of a typical Crouch gait.** In the left panel it is shown the hip and knee flexion-extension patterns for a gait cycle, while the right panel illustrates animations of the articulated model. Click here for file

Additional file 2 **Simulation of a typical Parkinsonian gait.** In the left panel it is shown the hip and knee flexion-extension patterns for a gait cycle, while the right panel illustrates animations of the articulated model. Click here for file

Additional file 3 **Simulation of a typical Normal gait.** In the left panel it is shown the hip and knee flexion-extension patterns for a gait cycle, while the right panel illustrates animations of the articulated model. Click here for file
